# Chemical Composition and In Vitro Antibiofilm Action of *Varronia curassavica* Jacq. (Boraginaceae) Essential Oil: A Promising Natural Agent Against Bacterial Infections

**DOI:** 10.1002/cbdv.202500924

**Published:** 2025-06-25

**Authors:** José Thyálisson da Costa Silva, Gabriel Gonçalves Alencar, Nara Juliana Santos Araújo, Lariza Leisla Leandro Nascimento, Saulo Almeida Menezes, Dhenes Ferreira Antunes, Adrielle Rodrigues Costa, Murilo Felipe Felício, Cicero dos Santos Leandro, Luis Pereira de Morais, Marcos Aurélio Figueirêdo dos Santos, Severino Denicio Gonçalves de Sousa, Julimery Gonçalves Ferreira Macedo, João Pereira da Silva Junior, Ana Carolina Ferreira Araujo, Maísa Freire Cartaxo Pires de Sá, Jacqueline Cosmo Andrade‐Pinheiro, Henrique Douglas Melo Coutinho, José Weverton Almeida‐Bezerra

**Affiliations:** ^1^ Department of Biological Sciences Regional University of Cariri—URCA Crato Brazil; ^2^ Federal University of Cariri—UFCA Crato Brazil; ^3^ Department of Biological Chemistry Regional University of Cariri—URCA Crato Brazil; ^4^ Biotechnology Center Federal University of Rio Grande do Sul—UFRGS Porto Alegre Brazil; ^5^ Department of Basic Life Sciences Juiz de Fora Federal University—UFJF Governador Valadares Brazil; ^6^ Federal University of Paraíba—UFPB João Pessoa Paraíba Brazil

**Keywords:** *Bacterial infections*, biofilm eradication, biofilm formation, GC–MS analysis, monoterpenes

## Abstract

Antimicrobial resistance is an increasing threat to public health, with alarming estimates of mortality rates. In this context, the clinical properties of *Varronia curassavica* Jacq. are highlighted due to its biological and pharmacological activities. This study aims to analyze the phytochemical composition of the essential oil from *V. curassavica* (EOVC) and its antibiofilm activity against Gram‐positive and Gram‐negative strains. The essential oil was extracted from the leaves using hydrodistillation and analyzed by gas chromatography–mass spectrometry (GC–MS), phytochemical analysis of EOVC identified 97.36% of the total composition, predominantly composed of monoterpenes (α‐pinene at 44.46%) and sesquiterpenes (β‐caryophyllene at 21.82%). Antibiofilm tests were performed on five bacterial strains (both Gram‐positive and Gram‐negative) to evaluate the ability to inhibit and eradicate biofilms, with statistical analysis of the results. Antibiofilm formation tests demonstrated that EOVC exhibited efficacy against *Staphylococcus aureus* and *Staphylococcus epidermidis*, with minimum inhibitory concentrations (MIC) of 1.024 and 10.240 µg/mL, respectively, values comparable to those observed for the reference antibiotic. In addition, at high concentrations (10 × MIC), EOVC inhibited biofilm development by *Streptococcus mutans* and *Enterococcus faecalis*, achieving performance similar to that of gentamicin. In biofilm eradication assays, EOVC showed effective activity against *E. faecalis*, *S. epidermidis*, and *S. aureus* at concentrations of 10.240 µg/mL, equivalent to gentamicin. However, resistance was found in strains of *Pseudomonas aeruginosa* and *S. mutans*, with *P. aeruginosa* showing the highest level of resistance to EOVC.

## Graphical abstract

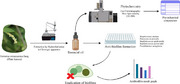

## 1 Introduction

Antimicrobial resistance is a growing threat to public health, and it is estimated that, if adequate measures are not adopted, the number of related deaths could exceed 10 million per year by 2050, surpassing deaths from cancer. This phenomenon manifests itself when microorganisms, including bacteria, fungi, parasites, and viruses, develop mechanisms that make them insensitive to previously effective drugs [1, 2, 3]. It can be classified as intrinsic, when it is naturally present in the species, or acquired, resulting from genetic mutations or the acquisition of resistance genes resulting from transfers [4].

Bacterial resistance is often associated with the formation of biofilms, multicellular structures developed in response to adverse conditions and characterized by high drug tolerance [5]. These biofilms can be made up of microorganisms of the same species or of different species, organized within a polymeric extracellular matrix and adhered to biotic or abiotic surfaces. This organization confers protection against antimicrobial agents, contributing to the persistence of chronic infections, since microorganisms in biofilm exhibit multiple resistance to antibiotics [6, 7].

There is a need for appropriate clinical interventions and therapies is observed, considering that the application of ineffective measures can result in serious consequences, leading to a scenario similar to that of the pre‐antibiotic era, where previously treatable diseases could result in a significant increase in deaths, highlighting that bacteria such as *Pseudomonas aeruginosa*, are predominantly responsible for infections in hospital settings [8, 9, 10]. However, it is already relatively pointed out that natural products, such as essential oils, have demonstrated high efficiency in antibacterial and antibiofilm activity against multidrug‐resistant bacteria [11, 12, 13, 14].

Medicinal plants have been explored for their potential in ethnopharmacology, standing out as an important natural source of biologically active compounds. These compounds offer innovative and alternative applications in treating diseases caused by microorganisms, with notable activities such as anticancer, antidiabetic, antioxidant, and antimicrobial. Moreover, there is evidence that the combination of these compounds may result in synergistic interactions, increasing efficacy against bacteria and fungi [15, 16, 17, 18].

Research on essential oils has shown significant clinical and pharmacological relevance due to their antibacterial, anti‐virulence, antifungal, anti‐inflammatory, and antioxidant properties, all of which have been clinically proven [11, 19, 20]. In this context, the clinical properties of *Varronia curassavica* Jacq. (syn. *Cordia verbenacea* A. DC. and *Cordia curassavica* (Jacq) Roemer & Schultes) stand out, as this plant is known for its anti‐inflammatory, analgesic, and antimicrobial activities. Its plant material is used in various forms, including extracts, essential oils, and infusions [21, 22, 23, 24].

From this perspective, research into the pharmacological properties of *V. curassavica* has revealed significant potential, particularly its antimicrobial activity. The plant has been shown to be effective against pathogenic microorganisms, especially in combating biofilms, a form of resistance that contributes to the increase in antimicrobial resistance. This study aims to quantify and analyze the phytochemical compounds of *V. curassavica* essential oil (EOVC), as well as to investigate its antibiofilm activity (biofilm prevention and eradication) against different Gram‐positive and Gram‐negative strains.

## 2 Materials and Methods

### 2.1 Plant Material

**FIGURE 1:**
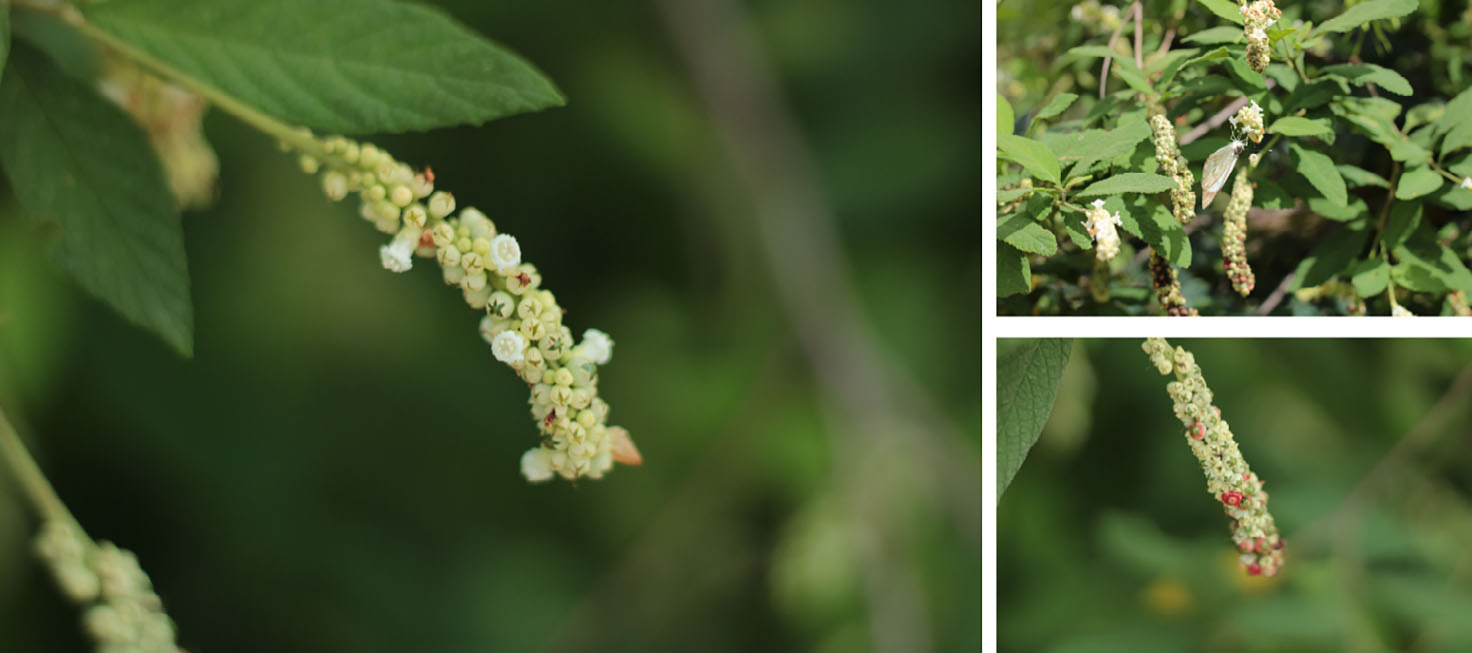
*Varronia curassavica* species, collected in the municipality of Jardim, Brazil. Inflorescence, fruits, and leaves.

The leaves of *V. curassavica* (Figure 1) were obtained in the municipality of Jardim, Ceará, Brazil, at coordinates −7.554917 W and −39.306611 S, during January (2022). A sample of the material was collected and deposited at the Dárdano de Andrade Lima Herbarium (HCDAL) of the Regional University of Cariri (URCA) under voucher number 15.291. The research was registered in the National System for the Management of Genetic Heritage and Associated Traditional Knowledge (SisGen) under the code AEFC723 and in the Biodiversity Authorization and Information System (SisBio) under number 82789‐1.

### 2.2 Extraction of Essential Oil

The essential oil was obtained from fresh *V. curassavica* leaves through the hydrodistillation method, using a Clevenger‐type apparatus. The leaves were dried naturally and crushed by hand, then subjected to a 5 L round‐bottomed flask containing 2 L of boiling water for a period of 2 h. The steam was then condensed, and the essential oil was separated from the aqueous phase. The collected oil was stored in amber flasks and refrigerated at 4°C. The essential oil obtained from the leaves of *V. curassavica* showed a yield of 1.6%.

### 2.3 Gas Chromatography–Mass Spectrometry

The gas chromatography–mass spectrometry (GC–MS) technique was applied to conduct the phytochemical analysis of the EOVC. The equipment used was the Agilent Technologies AutoSystem XL GC–MS system, operating in electron ionization mode at 70 eV. Two capillary columns were employed: HP 5MS (30 m × 0.35 mm; film thickness 0.50 mm) and HP Innowax (30 m × 0.32 mm; film thickness 0.50 mm). The system included a split/splitless injector set at 220°C and was connected to a flame ionization detector (FID). The temperature program was set from 60°C (1 min) to 180°C, with a heating rate of 3°C/min; the detector temperature was maintained at 220°C. Helium was used as the carrier gas at a flow rate of 1.0 mL/min. The injected volume of EOVC was 1 µL, diluted in chloroform at a 1:10 ratio. Replicated samples were analyzed, and the relative concentrations of the components were determined based on the chromatogram peak areas, without applying correction factors.

### 2.4 Antibiofilm Activity

#### 2.4.1 Strains, Culture Medium, Inocula, and Drug

The bacterial strains analyzed included *Streptococcus mutans* (INCQS 00446, ATCC 25175), *Enterococcus faecalis* (INCQS 00018, ATCC 14506), *Staphylococcus epidermidis* (INCQS 00016, ATCC 12228), *Staphylococcus aureus* (ATCC 25923), and *P. aeruginosa* (ATCC 9027). The microorganisms were obtained from the Microbiology and Molecular Biology Laboratory at the Regional University of Cariri (URCA) and the Oswaldo Cruz Foundation (FIOCRUZ). The strains were cultured on brain heart infusion (BHI) agar, and incubation was performed for 24 h at 37°C in an incubator.

After this period, a fraction of the cells was removed and diluted in a 0.85% NaCl solution, with the suspensions adjusted to a concentration of 5 × 10^5^ CFU/mL [25]. The EOVC was weighed, dissolved in dimethyl sulfoxide (DMSO), and then diluted in sterile water to obtain concentrations of 1.024 and 10.024 µg/mL. Gentamicin was used as the standard antibiofilm drug in the tests. DMSO was used as the solvent for the substances.

#### 2.4.2 Biofilm Formation Assay

The evaluation of biofilm formation by the isolates was carried out in microtiter plates using the crystal violet method with adaptations as described by Stepanovic et al. [26] and Andrade et al. [27]. A total of 160 µL of culture medium (BHI), 20 µL of distilled water, and 20 µL of bacterial inoculum adjusted to 1.5 × 10^8^ CFU/mL were added to the microtiter plates. For sterility control, the bacterial inoculum was replaced with distilled water. After incubating at 37°C for 24 h in a biological incubator, the plates were washed three times with 0.9% saline solution and then incubated at 55°C. Afterward, 200 µL of crystal violet were added for 15 min, and the plates were washed with distilled water, followed by elution with 100% ethanol and absorbance reading at 492 nm.

#### 2.4.3 Antibiofilm Formation

To evaluate the inhibition of biofilm formation by EOVC, 20 µL of MIC concentrations (minimum inhibitory concentration, 1.024 µg/mL) and MIC × 10 (10.024 µg/mL) of EOVC were added to microtiter plates, along with 20 µL of bacterial inoculum (1.5 × 10^8^ CFU/mL) and 160 µL of growth medium. NaCl 0.85% was used as the growth and sterility control. The plates were incubated for 24 h at 37°C. After incubation, planktonic cells were removed by washing three times with 0.9% saline solution. To fix the biofilm, the plates were incubated at 55°C for 60 min, and the biofilm was stained with 0.4% crystal violet solution for 15 min. The plates were then washed three times with saline solution and eluted with absolute ethanol for optical density reading at 492 nm. Antibiofilm activity was calculated by comparing the results with the growth control.

#### 2.4.4 Biofilm Eradication

After 48 h of preparation, the biofilms were treated with 20 µL of different concentrations of EOVC and gentamicin, with controls maintained in the microdilution plates. The plates were incubated at 37°C for 24 h, followed by the removal of the excess solution. Subsequently, the plates were washed three times with saline solution, incubated at 55°C for 60 min, and stained with 0.4% crystal violet for 15 min. The dye was then removed with saline solution and eluted with 100% ethanol. The absorbance was read at 492 nm [27].

### 2.5 Statistical Analysis

For statistical analysis, the GraphPad Prism software, version 6 (GraphPad Software Inc., San Diego, CA, USA), was used. The data were evaluated using the arithmetic mean of triplicates for each tested concentration and analyzed by two‐way ANOVA (*p* < 0.05; *p < 0.1; ****p < 0.0001), followed by Bonferroni post hoc test.

## 3 Results

### 3.1 Phytochemical Analysis of Essential Oil (GC–MS)

The phytochemical investigation of EOVC using GC–MS resulted in the identification of a total of 11 chemical compounds, which correspond to 97.36% of the essential oil composition. The largest portion of EOVC consists of hydrocarbon monoterpenes (46.3%), followed by hydrocarbon sesquiterpenes (42.35%). The major identified compounds were α‐pinene (44.46%), followed by β‐caryophyllene (sesquiterpene, 21.82%) and bicyclogermacrene (sesquiterpene, 12.77%), as presented in Table 1.

**TABLE 1:** Chemical composition (%) of the essential oil of *Varronia curassavica* (EOVC).

Components	RI	Molecular formula	(%)
Α‐Pinene	976	C_10_H_16_	43.46
β‐Pinene	980	C_10_H_16_	2.84
β‐Elemene	1375	C_15_H_24_	0.95
β‐Caryophyllene	1428	C_15_H_24_	21.82
α‐Humulene	1460	C_15_H_24_	3.15
Zingiberene	1492	C_15_H_24_	0.92
Bicyclogermacrene	1496	C_15_H_24_	12.77
*cis*‐α‐Bisabolene	1778	C_15_H_24_	2.74
Nerolidol	1961	C_15_H_26_O	4.12
Caryophyllene oxide	2023	C_15_H_24_O	1.71
Juniper camphor	2205	C_15_H_26_O	0.51
Hydrocarbon monoterpene		46.3	
Hydrocarbon sesquiterpene		42.35	
Oxygenated sesquiterpene		6.34	
Total		94.99	

Abbreviation: RI, retention index.

### 3.2 Bacterial Antibiofilm Activity

**FIGURE 2:**
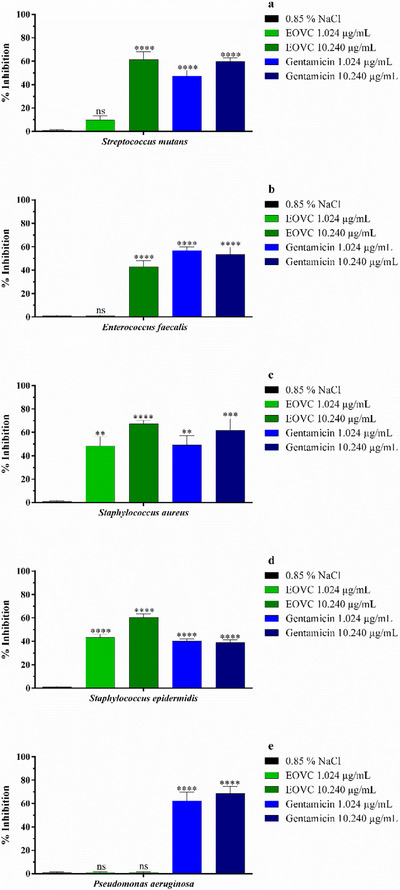
Antibiofilm formation activity of the essential oil from *Varronia curassavica* (EOVC) and the antibiotic gentamicin against *Streptococcus mutans* (A), *Enterococcus faecalis* (B), *Staphylococcus aureus* (C), *Staphylococcus epidermidis* (D), and *Pseudomonas aeruginosa* (E). **p < 0.01; ***p < 0.001; ****p < 0.0001; ns, not significant.

The evaluation of the anti‐bacterial biofilm formation activity of EOVC (Figure 2) and gentamicin showed different behaviors at concentrations corresponding to MIC (1.024 µg/mL) and MIC × 10 (10.240 µg/mL). At a concentration of 1.024 µg/mL, it was observed that only strains of *S. epidermidis* showed higher sensitivity, with significant inhibition attributed to EOVC, followed by strains of *S. aureus*. On the other hand, at a concentration of 10.240 µg/mL, the strains of *S. mutans*, *S. aureus*, and *S. epidermidis* showed high inhibition of biofilm formation by EOVC, and this activity is comparable to that observed with gentamicin, a drug used as a positive control. This effect can be associated with the presence of bioactive molecules in the essential oil (Table 1).

**FIGURE 3:**
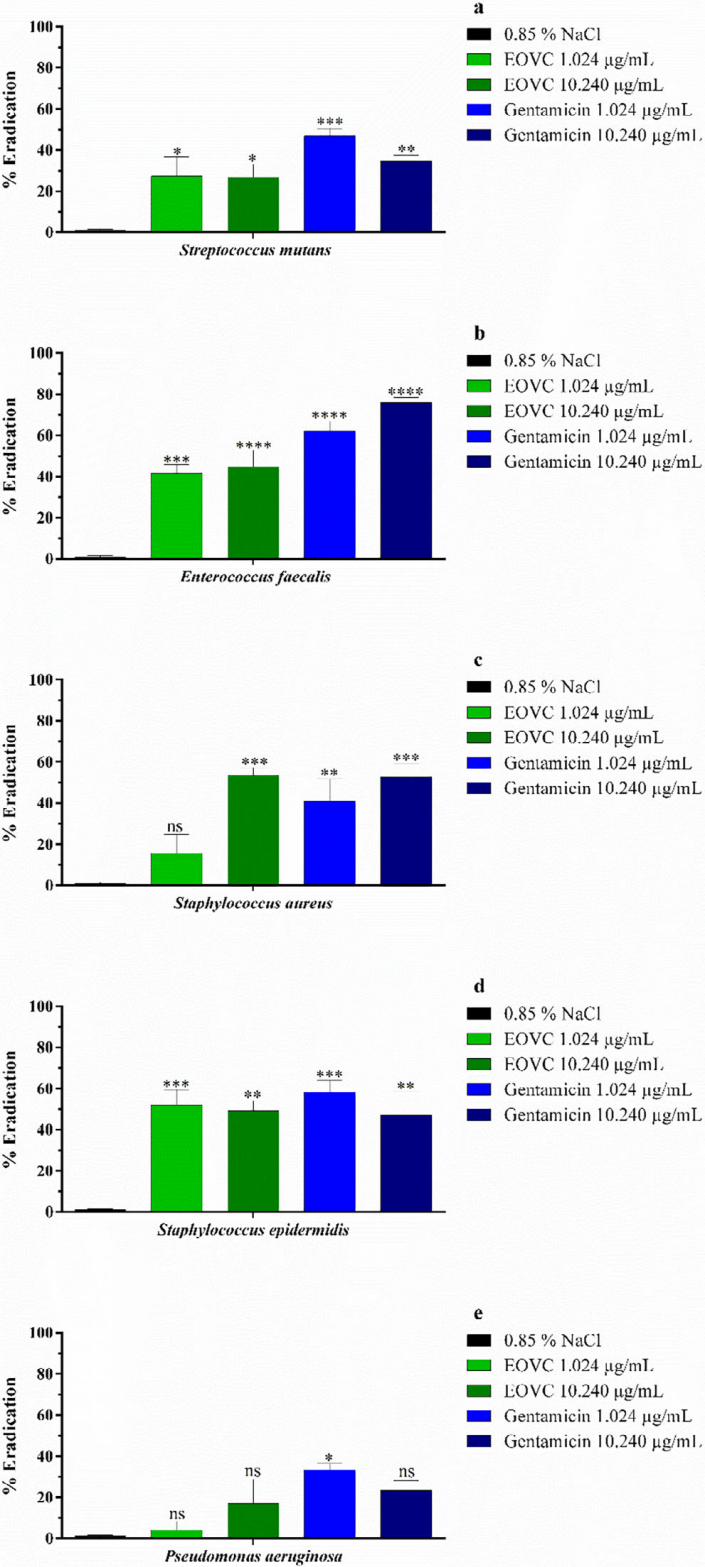
Biofilm eradication activity of the essential oil from *Varronia curassavica* (EOVC) and the antibiotic gentamicin against *Streptococcus mutans* (A), *Enterococcus faecalis* (B), *Staphylococcus aureus* (C), *Staphylococcus epidermidis* (D), and *Pseudomonas aeruginosa* (E). *p < 0.05; **p < 0.01; ***p < 0.001; ****p < 0.0001. ns, not significant.

The results obtained for the eradication of bacterial biofilm by EOVC (Figure 3) revealed an action profile different from that observed in Figure 2. At a concentration of 1.024 µg/mL, relevant activity was detected against the strains of *E. faecalis* and *S. epidermidis*, the latter being the most susceptible, with performance similar to that of gentamicin (Figure 3D). On the other hand, at the concentration of 10.240 µg/mL, no significant changes were observed, except for the strains of *S. aureus* and *E. faecalis*, which showed significant inhibition. In addition, the analyses performed with *P. aeruginosa* strains demonstrated resistance both in the evaluations of formation (Figure 2E) and eradication (Figure 3E) of the biofilm by EOVC, at both concentrations tested (MIC and MIC × 10).

## 4 Discussion

The phytochemical investigation of the EOVC has been widely studied by the scientific community due to the presence of various bioactive compounds such as α‐pinene, β‐caryophyllene, and bicyclogermacrene, which confirm the results obtained in this study. Previous studies have identified these compounds as predominant, with relative concentrations ranging from 0.36% to 58.86% for α‐pinene, 5.17% to 27.4% for β‐caryophyllene, and 0.21% to 32.16% for bicyclogermacrene in the total composition of the essential oil [28, 29, 30, 31, 32, 33, 34, 35, 36, 37, 38, 39, 40, 41, 42].

Previous research has reported that the compound caryophyllene oxide was present at concentrations of 15.02 ± 23.12% [43], and shyobunol was detected at concentrations between 24.24 ± 27.46% [44]. The observed variations in the concentrations and yields of these compounds are associated with various abiotic and biotic factors, such as the age of the plant [41], irradiance, which influences the number of glandular trichomes [43], differences in collection sites [36, 38], ultrasonic drying pretreatment [30], as well as seasonal variations and harvest times [39].

Therefore, it is crucial to establish standards for reproducibility in biological tests and standardize the collection of biological material, considering factors such as location, season, date, and time [45]. These aspects are essential for the effective application of medicinal plants as antimicrobials, as such variations directly impact the biological and pharmacological actions of *V. curassavica*, including activities such as anti‐inflammatory action [46] and synergisms that enhance antifungal [29] and antibacterial effects [37], particularly against Gram‐negative and Gram‐positive bacterial strains, such as *S. aureus* (MIC 64 µg/mL) [34].

The literature reports that essential oils act as antibiofilm agents and have the potential to enhance the efficacy of antibiotics, emerging as a promising strategy in combating antimicrobial resistance [11]. They affect biofilm morphology, separation, destruction, and development, as observed in Gram‐positive bacteria like *Alicyclobacillus acidoterrestris*, *S. epidermidis*, and *S. mutans*, with this action potentially linked to the presence of monoterpenes and sesquiterpenes [47, 48, 49].

In the genus *Varronia*, antibiofilm activity is observed in the essential oil of *V. dardani*, which is primarily composed of sesquiterpenes and inhibits biofilm formation while increasing the susceptibility of strains like *E. faecalis* and *Streptococcus salivarius* to antibiotics [50]. Góis et al. [51] partially indicates that the genus *Varronia* (e.g., *V. globosa*) has potential for applications against biofilms formed by Gram‐positive bacteria like *Enterococcus faecium*. The antibiofilm activity of *V. curassavica* described in the literature is evidenced by the inhibitory action against *E. faecalis* and *Actinomyces israelii*, with emphasis on the hexane fraction, which promoted the elimination of *A. israelii* biofilms (MIC of 500 µg/mL) [52].

In addition, the EOVC exhibited strong antibiofilm activity against *Xanthomonas campestris* pv. *campestris*, with significant inhibition of biofilm formation at concentrations equivalent to 2 × and 1 × MIC, as well as efficacy observed at subinhibitory concentrations. These effects were confirmed through crystal violet staining and scanning electron microscopy analyses. The EOVC not only suppressed planktonic growth but also inhibited biofilm formation, reinforcing its potential as a natural antimicrobial agent. Furthermore, EOVC combinations showed additive effects in 70% of the evaluated combinations and synergistic effects in specific cases, indicating that these formulations may enhance bactericidal efficacy and offer sustainable alternatives for the control of *X. campestris* [37, 53].

The data obtained in this study suggest that EOVC has potential in controlling bacterial biofilms formed by Gram‐positive strains, with this activity attributed to the presence of bioactive terpenes such as α‐pinene. This monoterpene, widely identified in essential oils, possesses various biological properties, including antimicrobial and antibiofilm effects [54, 55]. In a study conducted by Sieniawska et al. [33], α‐pinene exhibited a MIC of 0.625 mg/mL against *S. epidermidis*, both in its planktonic form and during biofilm formation. Similar results report activity against *S. mutans* through the essential oil of *Pistacia vera* L. (composed of 91.25% α‐pinene) [56]. Moreover, its exposure reduces bacterial viability [57].

The antibacterial efficacy of α‐pinene was further confirmed against *S. aureus*, with a MIC of 128 µg/mL [58]. The essential oil of *Pistacia atlantica*, containing 93.17% α‐pinene, demonstrated potent activity against *Helicobacter pylori*, including strains resistant to metronidazole, reinforcing the potential of this phytoconstituent for antimicrobial applications [59]. The bacterial kill kinetics indicated that concentrations between 1.25 and 2.5 µL/mL were effective in eliminating *Escherichia coli* ATCC 25922 colonies within a 2‐h period [60].

β‐Caryophyllene has stood out for its effectiveness in combating bacterial strains, including *S. aureus*, *S. mutans*, *L. monocytogenes*, and *Salmonella typhimurium*, significantly inhibiting bacterial adhesion and biofilm formation. This activity is enhanced when the compound is used in combination with other bioactives, such as linalool, cinnamaldehyde, and eugenol, demonstrating synergistic action through the modulation of gene expression related to cell adhesion and polysaccharide capsule production [61, 62, 63]. Evidence suggests that this effect may be associated with interactions with proteins crucial for biofilm formation, such as CrtM and SarA, leading to alterations in their functional conformation and, consequently, their activity [64].

This sesquiterpene is widely distributed in essential oils from different plant species and is recognized for its antimicrobial activity. Literature data indicate its action against *S. aureus* and *P. aeruginosa* strains, as observed in the essential oil of *Vernonia remotiflora* Rich., whose volatile composition contains more than 40% β‐caryophyllene [65]. Moreover, in vitro and in vivo studies have demonstrated its activity against *H. pylori*, highlighting its therapeutic potential in gastroduodenal infections [66].

Several compounds present in the essential oil, such as β‐pinene, β‐elemene, α‐humulene, zingiberene, bicyclogermacrene, nerolidol, and β‐caryophyllene oxide, have demonstrated significant antimicrobial and antibiofilm activity (Table 2), emerging as promising agents in the fight against infections caused by bacteria and fungi, including resistant and biofilm‐forming strains. β‐Pinene exhibits antimicrobial activity against bacteria and fungi, with effective antibiofilm effects against *Candida* species [67, 68]. β‐Elemene shows activity against *Mycobacterium tuberculosis* [33], while α‐humulene stands out for its action against *Bacteroides fragilis*, inhibiting both biofilm formation and the expression of efflux genes [69].

**TABLE 2:** Phytochemical composition and bioproperty evidence of *Varronia curassavica e*ssential oil compounds.

	Components	Biological properties	Reference
1	α‐Pinene	Antimicrobial and antibiofilm activity	[33, 54, 55, 56]
2	β‐Pinene	Antimicrobial and antibiofilm activity	[67, 68]
3	β‐Elemene	Antibacterial activity	[33]
4	β‐Caryophyllene	Antimicrobial and antibiofilm activity	[61, 62, 63, 65]
5	α‐Humulene	Antibacterial and antibiofilm activity	[69]
6	Zingiberene	Antibacterial activity	[70]
7	Bicyclogermacrene	Antimicrobial activity	[71]
8	*cis*‐α‐Bisabolene	—	—
9	Nerolidol	Antimicrobial and antibiofilm activity	[72, 73]
10	Caryophyllene oxide	Antibiofilm activity	[74]
11	Juniper camphor	—	—

Zingiberene, which is the major compound in *Senecio selloi* Spreng DC. (54%), demonstrated selective activity against *Bacillus subtilis* ATCC 6633 [70]. Bicyclogermacrene acts as an antimicrobial adjuvant, especially when combined with β‐caryophyllene [71]. Nerolidol, in turn, exhibits a broad antimicrobial spectrum and potent inhibition of biofilm formation in *S. aureus*, including multidrug‐resistant strains, reinforcing the potential of these compounds as natural alternatives for the development of antimicrobial agents and modulators of microbial virulence [72, 73]. Finally, β‐caryophyllene oxide proved effective in inhibiting the formation and maturation of *C. albicans* biofilms, with low cytotoxicity [74].

## 5 Conclusion

The results obtained in this study highlight the potential of the EOVC as a promising alternative in the fight against bacterial biofilms, especially those formed by Gram‐positive bacteria, such as *S. epidermidis*, which demonstrated high sensitivity to the concentration of 1.024 µg/mL, comparable to gentamicin. Phytochemical analysis by GC–MS confirmed the presence of most compounds with recognized antimicrobial activity, such as the monoterpene α‐pinene and the sesquiterpene caryophyllene. These findings reinforce the relevance of the essential oil as a natural therapeutic agent.

Thus, EOVC represents a promising therapeutic alternative, especially in the face of the growing challenge of microbial resistance. Its potential application in topical formulations can contribute significantly to the reduction of infections associated with these devices, promoting better clinical outcomes. From a socioeconomic perspective, EOVC, obtained from a widely available medicinal plant, represents a sustainable and low‐cost strategy, particularly advantageous for communities with restricted access to synthetic medicines. In addition, valuing the cultivation and local processing of *V. curassavica* can boost regional economic development, strengthening family farming and encouraging the preservation of traditional knowledge linked to popular medicine.

This study highlights the potential of *EOVC* as a bacterial antibiofilm agent, effective against both Gram‐positive and Gram‐negative bacterial strains. However, the study has limitations in elucidating the mechanisms of action of the extract against microbial resistance, especially in biofilms. It is essential to carry out both in vitro and in vivo, safety evaluations to enable future applications. In addition, in silico analyses, such as molecular docking, are necessary to identify possible molecular targets. Further research should also cover toxicity testing, including cytotoxicity and genotoxicity in mammalian cell lines.

## Conflicts of Interest

The authors declare no conflicts of interest.

## Data Availability

The data that support the findings of this study are available from the corresponding author upon reasonable request.
